# The role of cigarette smoke-induced epigenetic alterations in inflammation

**DOI:** 10.1186/s13072-019-0311-8

**Published:** 2019-11-11

**Authors:** Dandan Zong, Xiangming Liu, Jinhua Li, Ruoyun Ouyang, Ping Chen

**Affiliations:** 1 Department of Respiratory and Critical Care Medicine, The Second Xiangya Hospital, Central South University, Changsha, 410011 Hunan China; 2 Research Unit of Respiratory Disease, Central South University, Changsha, 410011 Hunan China

**Keywords:** Epigenetic, DNA methylation, Histone modification, miRNA, LncRNA, Inflammation

## Abstract

**Background:**

Exposure to cigarette smoke (CS) is a major threat to human health worldwide. It is well established that smoking increases the risk of respiratory diseases, cardiovascular diseases and different forms of cancer, including lung, liver, and colon. CS-triggered inflammation is considered to play a central role in various pathologies by a mechanism that stimulates the release of pro-inflammatory cytokines. During this process, epigenetic alterations are known to play important roles in the specificity and duration of gene transcription.

**Main text:**

Epigenetic alterations include three major modifications: DNA modifications via methylation; various posttranslational modifications of histones, namely, methylation, acetylation, phosphorylation, and ubiquitination; and non-coding RNA sequences. These modifications work in concert to regulate gene transcription in a heritable fashion. The enzymes that regulate these epigenetic modifications can be activated by smoking, which further mediates the expression of multiple inflammatory genes. In this review, we summarize the current knowledge on the epigenetic alterations triggered by CS and assess how such alterations may affect smoking-mediated inflammatory responses.

**Conclusion:**

The recognition of the molecular mechanisms of the epigenetic changes in abnormal inflammation is expected to contribute to the understanding of the pathophysiology of CS-related diseases such that novel epigenetic therapies may be identified in the near future.

## Background

Cigarette smoke (CS) presents a significant risk factor related to various diseases, such as cardiovascular diseases [1], chronic obstructive pulmonary disease (COPD) [2], and cancers [3]. Numerous chemical substances inside tobaccos have toxic effects, including polycyclic aromatic hydrocarbons (benzo[a]pyrene), *N*-nitrosamines, heavy metals (nickel, cadmium, chromium and arsenic), alkaloids (nicotine and its major metabolite, cotinine) and aromatic amines. The pathogenic mechanisms of CS include inflammation and immune changes, genetic alterations, oxidative damage, endothelial dysfunction, cell senescence, etc. [4]. However, tobacco use confers long-term risk of diseases, even decades after cessation, that are not well understood [5]. Protein-coding genes cannot account for all environmental diseases. Recently, epigenetic changes have been proposed as possible alternative causes of some diseases.

**Fig. 1:**
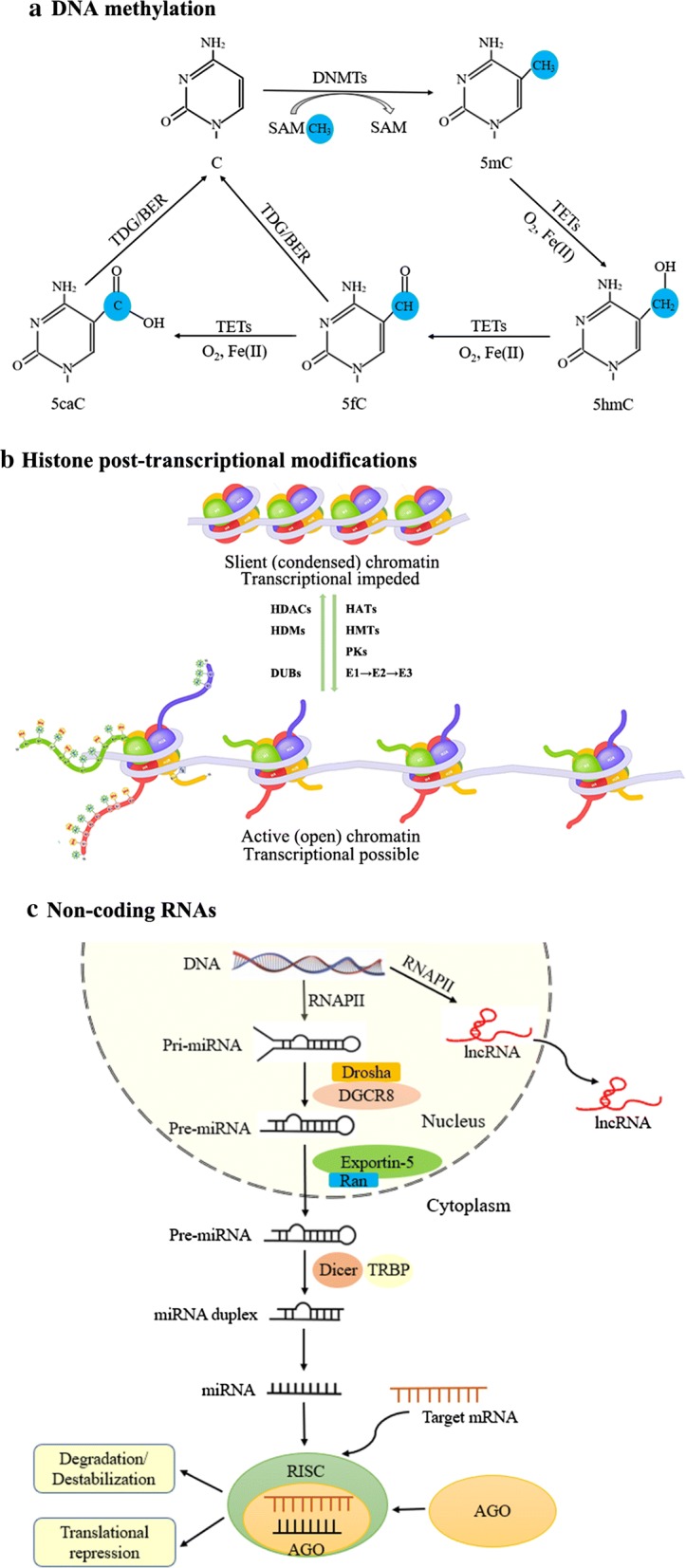
Epigenetic mechanisms. **a** DNA methylation. DNA methylation is catalyzed by DNA methyl transferases (DNMTs). DNA methylation involves the covalent transfer of a methyl group from *S*-adenosyl methionine (SAM) to 5′ position of cytosine residues in CG dinucleotides. DNA demethylation involves ten-eleven translocation (TET) proteins. TETs initiate DNA demethylation by oxidizing 5-methylcytosine (5mC) to 5-hydroxymethylcytosine (5hmC), which can be further oxidized to 5-formylcytosine (5fC) and 5-carboxylcytosine (5caC). 5fC and 5caC can be recognized and excised by thymine DNA glycosylase (TDG), and the residual abasic site is repaired as unmodified C by base excision repair (BER) pathway to complete “active” demethylation. Furthermore, oxygen and Fe(II) are indispensable for the TET enzymes to perform the successive oxidation of 5mC, 5hmC and 5fC. **b** Histone posttranscriptional modifications. Histone modification is regulated by opposing enzymes. Histone acetylation is mediated by histone acetyltransferases (HATs) and deacetylation by histone deacetylases (HDACs). Methylation is mediated by histone methyltransferase (HMTs) and demethylation by histone demethylases (HDMs). Phosphorylation is mediated by phosphorylase kinases (PKs). Ubiquitination is carried out through three main enzymatic reactions performed stepwise by ubiquitin-activating enzyme (E1), ubiquitin-conjugating enzyme (E2), and ubiquitin ligase (E3) consecutively; deubiquitination is mediated by deubiquitinating enzymes (DUBs). **c** The biogenesis of miRNA and lncRNA. The miRNA is transcribed to create the primary miRNA (pri‐miRNA) by RNA polymerase II (RNAPII). Following cleaved by the Drosha and DRCG8, precursor miRNA (pre‐miRNA) is exported from the nucleus by exportin 5. Then, Dicer and TAR RNA‐binding protein (TRBP) will further process the molecule and form a double-stranded miRNA: miRNA duplex. One strand of the duplex, together with the argonaute (AGO) protein and the target messenger RNA, is incorporated in the RNA-induced silencing complex (RISC) and subsequently targets mRNAs for degradation or translational repression. The lncRNA is transcribed mostly by RNAPII and its biogenesis process is similar to miRNA

Epigenetic research is becoming a rapidly developing and exciting area in biology. Epigenetics is usually defined as heritable alterations in gene expression patterns that are not directly caused by modifications encoded in the nucleotide genome sequence but are caused by posttranslational modifications in DNA and histone proteins and by the regulation of non-coding RNAs (ncRNAs) [6]. The epigenetic mechanisms are shown in Fig. 1. DNA is methylated by the transfer of a methyl group from *S*-adenosyl-l-methionine to covalently bind to the cytosines in CpG dinucleotides. DNA hypermethylation always leads to transcriptional suppression and decreased gene expression, while DNA hypomethylation influences chromosome stability or enhanced aneuploidy [7]. Eukaryotic chromatin is made up of nucleosomes, which consist of ~ 147 bp of DNA wrapped around an octamer of two copies of four core histones (H2A, H2B, H3, and H4). Posttranslational modification of histones involves covalent modification of specific sites and residues that form specific three-dimensional arrangements of nucleosomes that control the accessibility to transcriptional factors to genes to induce gene expression. Histones are primarily modified at basic lysine and arginine residues, thereby marking active and inactive chromatin states [8]. Important histone modifications include acetylation, methylation, phosphorylation, and ubiquitination. NcRNAs, once thought to represent transcriptional noise, are now recognized as having important functions in cellular processes [9]. Indeed, only a small proportion of the human genome encodes protein (~ 2%); the majority of the genome is not translated into proteins and may instead code a range of ncRNA types (> 90% of the genome). These ncRNAs are classified into small ncRNAs, with transcript length of 200 or fewer nucleotides, and long ncRNAs (lncRNAs), with transcript lengths more of > 200 nucleotides [10]. Small RNAs include but are not limited to microRNAs (miRNAs), PIWI-interacting RNAs, small interfering RNAs, small nucleolar RNAs, and transfer RNAs. In recent years, growing evidence has suggested that ncRNAs, especially miRNAs and lncRNAs, play important roles in the pathogenesis of human diseases [11, 12].

Epigenetic regulation of gene and protein expression is carried out by a set of highly conserved processes that contribute to normal development and adaptation to changes in cellular and organ homeostasis [13]. It is very important to note that the changes caused by epigenetic modifications of DNA and/or histones and changes in ncRNA expression represent dynamitic information that is markedly influenced by environmental stimuli, such as CS, air pollution and dietary changes. Epigenetic dysregulation that leads to inappropriate gene expression or silence has been shown to play a significant role in the CS-related diseases. Moreover, it can combine with other CS-related molecular mechanisms, such as oxidative stress, inflammation, apoptosis, and senescence. Understanding the basic mechanisms induced by CS and the relationships among them is invaluable to our knowledge of cellular differentiation and genome programming and the connection of these relationships with diseases. Herein, we provide a comprehensive overview of the current research on how epigenetic alterations are combined with other pathogenic mechanisms induced by CS during the disease processes.

## The effect of cigarette smoke on epigenetics

### Cigarette smoke and DNA methylation

Alterations in DNA methylation have recently been suggested to be one possible mechanism potentially mediating CS-induced diseases [14]. Several epigenome-wide association studies based on different sample sizes, races and areas have confirmed that the altered DNA methylation at multiple CpG sites is induced by CS [15–23] (Table 1) and can lead to changes in gene transcription and increased susceptibility to diseases. A recent meta-analysis of genome-wide DNA methylation assessed nearly 16,000 blood-derived DNA samples from participants in 16 cohorts and the results showed that 7201 annotated genes associated with 18,760 CpGs, representing almost one-third of all known human genes, were differentially methylated in the current smokers versus the number found in the never smokers [5]. DNA methylation altered by tobacco smoke is a reversible gene regulatory modification. After smoking cessation, the majority of the differentially methylated CpG sites observed in the analysis of the current versus never smokers returned to the level of the never smokers within 5 years of smoking cessation. However, some of the methylated genes did not return to the level of the never smokers even after 30 years of smoking cessation [5], suggesting that CS may lead to lasting damage to human health. In addition to the current smokers, human DNA methylation at specific CpG sites can also be influenced by maternal smoking. Howe et al. [24] analyzed the methylomes of sorted cord blood CD4+ cells derived from 20 Hispanic white newborns. Of the newborns, 10 were exposed to maternal tobacco smoke in pregnancy and 10 were not exposed. They found that 10,381 CpGs were differentially methylated by CS exposure. Five-hundred and fifty-seven differentially methylated regions were overrepresented in important regulatory regions, including those of enhancers [24]. Joubert et al. [25, 26] demonstrated that maternal smoking affects DNA methylation in newborn cord blood if the mother smoked past 18 weeks into her pregnancy, whereas significant effects on methylation were not observed for mothers who quit smoking before 18 gestational weeks. Given that smoking is an established risk factor for many chronic diseases, these loci could have important applications as objective biomarkers of both current and lifetime smoking exposure and for quantifying risks of smoking-related diseases. As DNA methylation is mainly catalyzed by DNA methyltransferases (DNMTs), researchers found that *DNMT1* expression was significantly higher in the lung tissues of the smokers compared to the expression in the nonsmokers [27]. Furthermore, the inhibition of *DNMT1* can restore the expression of genes that had been suppressed by a CS condensate through demethylation [28]. Thus, CS can induce gene hypermethylation by upregulating *DNMT1*, which in turn leads to the downregulation of target genes.

**Table 1:** The changes of DNA methylation profiles affected by CS

Sample size	Population	Tissue for DNA methylation analysis	Smoking status	Main result	References	Year
596	Chinese population	Whole blood	Current smoke	318 CpG sites were differentially methylated due to cigarette smoking.	Zhu et al. [15]	2016
745	European women	Whole blood	Current smoke	461 CpG sites were aberrant methylated due to cigarette smoking. Of them, 448 CpG sites were hypomethylated	Guida et al. [16]	2015
			Former smoke	The methylation of 751 CpG sites were changed the recoded smoking status. Of them, 602 CpG sites reverted back to that of never smokers from up to 35 years after smoking cessation. 149 CpG sites remained differentially methylated > 35 years after smoking		
123	Arab population	Whole blood	Current smoke	Aberrant methylation was detected due to tobacco smoking. The exact number of CpG sites was not mentioned	Zaghlool et al. [17]	2015
111	African American women	Peripheral blood mononuclear cells	Current smoke	910 loci were found to be differentially methylated in smokers	Dogan et al. [18]	2014
192	South Asian and European men	Whole blood	Current smoke	29 CpG sites at 18 unique loci were differential methylated in smokers	Elliott et al. [19]	2014
180	Italian population	Peripheral white blood cells	Current smoke/former smoke	17 and 19 loci in the breast cancer and colon cancer were differentially methylated between smokers, former smokers and never smokers. In former smokers, methylation levels at AHRR, 2q37 and 6p21 loci returned to the levels of nonsmokers with increasing time from cessation and those who had smoked more intensively had methylation levels that were closer to that of current smokers	Shenker et al. [20]	2013
1793	Augsburg population	Whole blood	Current smoke/FORMER smoke	972 CpG sites were differentially methylated after smoking. Of which, 187 CpG sites were differentially methylated in current smokers.	Zeilinger et al. [21]	2013
98	African Americans	Lymphocyte cells	Current smoke	Aberrant methylation was detected due to tobacco smoking. The authors listed 30 most significant methylated CpG sites without mentioning the exact number of methylated CpG sites	Philibert et al. [22]	2013
972	African Americans	Peripheral leukocytes	Current smoke	15 autosomal DNAm sites were significantly associated with current smoking using a Bonferroni corrected *p* value of 0.05. 89 DNAm sites associated with current smoking with FDR *q* value less than 0.05	Sun et al. [23]	2013
20	Hispanic newborns	CD4+ cells from cord blood	In utero exposure to maternal tobacco smoke	10,381 CpG sites were differentially methylated by tobacco smoking. Of them, 557 differentially methylated regions were overrepresented in important regulatory regions, including enhancers	Howe et al. [24]	2019
1062	Norwegian newborns	Cord blood	In utero exposure to maternal tobacco smoke	Differential DNA methylation of 26 CpG sites mapped to 10 genes were found in newborns born to smoking mothers compared to nonsmoking mothers	Joubert et al. [25]	2012
1042	Norwegian newborns	Cord blood	In utero exposure to maternal tobacco smoke	Maternal smoking affected DNA methylation of 26 CpG sites that mapped to 10 genes in newborn cord blood if the mother smokes past 18 weeks in pregnancy, whereas significant effects on methylation were not observed for mothers that quit before 18 gestational weeks	Joubert et al. [26]	2014

### Cigarette smoke and histone posttranscriptional modifications

#### Cigarette smoke and histone acetylation

Previous studies reported that CS caused histone hyperacetylation in the lungs of both human smokers [29, 30], mouse models exposed to CS [31], and human bronchial epithelial (HBE) cells treated with cigarette smoke extract (CSE) in vitro [32]. It was found that active smoking may promote acetylation of histone H4, while ex smokers showed increased histone H3 acetylation [29]. Histone acetylation is a dynamic process that depends on the balance of two opposing types of enzymes: histone acetyltransferases (HATs), which add an acetyl group, and histone deacetylases (HDACs), which remove acetyl groups from conserved lysine residues and nonhistone proteins [33]. Current studies have shown that CS induces increased histone acetylation mainly by decreasing the activity and expression of HDACs [29–32]. Moreover, there is a significant and negative relationship between HDAC activity and smoking exposure levels [34]. In the lung tissues of smokers with COPD, the expression of *HDAC2*, *HDAC5*, *HDAC8* [29, 30], *HDAC7* [35] and *HDAC10* [36] was decreased significantly. An increased number of acetylated H3 and H4 proteins were shown to be associated with decreased expression of *HDAC1* [37], *HDAC2* and *HDAC4* [38] in CS-treated rat lungs.

In bronchial epithelial cells and alveolar macrophages exposed to CSE, the expression and activity of *HDAC3* were reported to be decreased [39, 40]. In contrast to that of other HDACs, cytoplasmic *HDAC6* expression is elevated in the lung tissues of chronic smokers with COPD, partly due to hypomethylation by *HDAC6* [41]. Different results were found by Borgas et al. [42], who showed that *HDAC6* protein levels were not changed in cultured lung endothelial cells exposed to CSE in vitro but significantly decreased in the lung tissues of mice exposed to CS for 3 weeks. Although there is a discrepancy regarding the impacts of CS on *HDAC6* protein levels, *HDAC6* may be activated both in vivo and in vitro through *HDAC6* phosphorylation at Ser-22 [42]. Sirtuin (*SIRT*)*1*-*7* belongs to the family of class III HDACs. It has been shown that the expression of *SIRT1* was decreased when exposed to CS both in vivo and in vitro [43–45]. Furthermore, a statistically positive correlation was found between *SIRT1* activity and two lung function parameters (FEV1 and FEV1/FVC) [43]. In the CS-induced hypertension mouse model, the expression of *SIRT3* was reduced, as was the hyperacetylation of superoxide dismutase 2, and superoxide dismutase 2 activity was inhibited, which promoted mitochondrial oxidative stress [46]. Other SIRTs, such as *SIRT4* [47], *SIRT5* [48] and *SIRT6* [49], were markedly downregulated when exposed to CS. In addition to its effect on HDACs, CS also alters the expression of HATs. In CS-treated bronchial epithelial cells, the expression of an HAT, cAMP-response element-binding protein (*CBP*/*p300*), was demonstrated to be increased and to induce a multitude of acetylating processes [50]. In short, decreased HDAC action and increased HAT action may account for CS-induced hyperacetylation.

#### Cigarette smoke and histone methylation

Previous researchers have reported that global levels of histone methylation were increased after CS exposure [32]. Both mono-and di-methylation of histone H3 residues (H3K27me2/3, H3K36me1/2, H3K56me2, and H3K79me1/2) and histone H4 residues (H4K20me1/2, H4R23me1, H4K31me2, H4R35me1/2, H4R36me1, H4R55me1 and H4K77me1) were increased in CS-exposed mouse lungs [32]. As the catalytic enzyme for histone methylation, histone methyltransferase (HMT) was also found to be regulated by CS. *PRMT6* is a nuclear enzyme that specially catalyzes di-methylation of R2 in histone H3 (H3R2me2a). In previous studies, we demonstrated that CS may decrease the protein abundance of *PRMT6* at the same time that H3R2me2a was decreased both in CS-exposed mouse lungs and in CSE-treated endothelial cells, which further influence cell apoptosis and inflammation [51, 52]. Enhancer of zeste homolog 2 (*EZH2*) is a specific HMT that catalyzes histone H3 lysine 27 tri-methylation (H3K27me3). It was shown that CSE can enhance the expression of *EZH2* and increase the level of H3K27me3, which epigenetically controlled gene transcription [53]. To date, few studies have been concerned with changes in histone methylation caused by CS. Due to the regulating effects of histone methylation on gene transcription, understanding the role of histone methylation and their associated HMTs will provide insights into the clinical treatment of smoking-related diseases.

#### Histone phosphorylation

Phosphorylation of histone has been reported to be affected by various stimuli, such as arsenite, nickel, ultraviolet B rays and CS [54]. Treatment with CS markedly induced the phosphorylation of histone H3 at serine 10 and 28 residues [54], as well as histone H2AX [55], in a dose- and time-dependent manner. A number of kinases, such as ribosomal S6 kinase (*RSK*) and mitogen- and stress-activated kinase (*MSK*), were found to phosphorylate histone H3 depending on the specific stimulation or stress [56]. Sundar et al. [57] showed that CSE medicated the activation of *MSK1*, which was associated with the phosphorylation of histone H3S10 and further promotes the expression of pro-inflammatory genes. Knocking down *MSK1* inhibited CSE-induced posttranslational modifications of histones and the transcription of pro-inflammatory genes [57]. MSKs are activated via a complex series of phosphorylation and autophosphorylation reactions by the extracellular signal-regulated kinase (*ERK*) and p38 mitogen-activated protein kinase (*p38 MAPK*) pathways [58]. A large number of studies have demonstrated that CS can activate the *ERK* and *p38* pathways [59–61]. Another MAPK subfamily, c-jun N-terminal kinase (*JNK*), which mediates part of the stress response, was shown to induce phosphorylation of H3S10 [62]. The finding that CS activates the *JNK* pathway has been supported by numerous studies [61, 63, 64]. Taken together, these findings indicate that CS can induce histone phosphorylation alterations through the activation of various kinases.

#### Cigarette smoke and histone ubiquitination

Extensive studies have confirmed that CS exposure contributes to the development of skeletal muscle atrophy, which occurs when the level of protein breakdown exceeds protein synthesis [65]. Wasting of the muscle tissue is believed to the result of increased ubiquitin–proteasome system (UPS) activity, which can be induced by CS [66, 67]. Kim et al. [68] confirmed that CS induced protein degradation of *Akt*, which plays a critical role in cell survival and proliferation, by the UPS. Ubiquitination is carried out through three main enzymatic reactions performed stepwise by ubiquitin-activating enzyme (E1), ubiquitin-conjugating enzyme (E2), and ubiquitin ligase (E3) consecutively [69]. Muscle atrophy F-box (*MAFbx*/*atrogin*-*1*) and muscle ring finger-1 protein (*MuRF1*) have been identified as two muscle-specific E3 ligases. Both these two E3 ligases play central roles in muscle atrophy. Baptista et al. [70] found that leucine supplementation administered to rats with immobilized hindlimbs attenuated soleus muscle mass loss and minimized the *MuRF1* and *MAFbx*/*atrogin*-*1* gene expression. Moreover, mice deficient in either *MAFbx* or *MuRF1* were protected from muscle atrophy, whereas overexpressed *MAFbx* in myotubes induced atrophy [71]. It has been shown that C2 myotubes exposed to CS upregulated *MAFbx*/*atrogin*-*1* and *MuRF1* by activating the *p38 MAPK* signaling pathway, which led to a reduction in myotube diameter and degradation of the main contractile proteins in a time- and dose-dependent manner [72]. Similar to its effect on the *p38 MAPK* pathway, CS exposure also resulted in nuclear factor kappa B (*NF*-*κB*) activation, which led to upregulated levels of *MuRF1* and reduced myotube diameter of myotubes and time-dependent degradation of the main contractile protein, myosin heavy chain [73]. In addition to the E3 ligases, other ubiquitin-specific proteases (USPs), such as *USP19*, were also observed to be increased in CS-induced muscle atrophy [74]. These results suggest that CS can induce skeletal muscle atrophy and degradation via the activation of the UPS. Mitochondrial E3 ubiquitin protein ligase 1 (*MUL1*) is a novel *Akt* ubiquitin E3 ligase. CS can noticeably elevate *MUL1* expression and *Akt* ubiquitination, while knocking down *MUL1* suppressed CS-induced *Akt* ubiquitination/degradation and inhibited the reduction in cytoplasmic *Akt* and *p*-*Akt* [75]. USP17 has been shown to be an E3 deubiquitinase that inhibits proteasome-mediated degradation of transcription factors [76]. It has been shown that *HDAC2* is excessively ubiquitinated and degraded in the proteasome, an action attributed to the low expression of *USP17* in CS-exposed airway epithelial cells and macrophages, while overexpressed *USP17* blocks the degradation of *HDAC2* induced by CS [77].

### Cigarette smoke and non-coding RNAs

#### Cigarette smoke and miRNAs

To date, more than 48,800 mature miRNAs have been reported in 271 species. Over 2650 human miRNAs are listed in the Sanger miRBase sequence database (ver. 22). Studies have revealed that miRNAs play key roles in regulating various physiological and pathological processes, such as cell growth, differentiation, proliferation, apoptosis, and organ development [78]. Approximately, 30% of known human protein-coding genes are predicted to be regulated by miRNAs [79]. Thus, an altered expression of miRNAs has been linked to various human diseases, and the profiles of tissue miRNAs suggest potential applications in diagnosis and prognosis. As an unstable single-stranded RNA, miRNAs can be easily influenced by environmental chemicals, and the effects of CS exposure on miRNA are among the most intensely studies areas of research. Recent studies have shown that exposure to CS leads to global alteration in miRNAs. Early in 2009, Izzotti et al. [80] first analyzed miRNA expression patterns in the lungs of rats exposed to environmental CS for 28 days. The researchers reported that, of the 484 miRNAs analyzed, 126 miRNAs (26.0%) were downregulated by at least twofold, and 24 miRNAs were downregulated more than threefold, and the difference between rats exposed to CS and sham group was significant. Only 7 miRNAs (1.4%) were upregulated. The same team also investigated the effects of CS exposure on miRNA expression in the lungs of mice [81]. Their findings were similar in that the miRNA alterations in the lungs of the mice were primarily downregulated. The dysregulation of miRNA expression was gender and age dependent. Moreover, a dose- and time-dependent effect of CS on miRNAs was also found by Izzotti and his colleagues [82, 83]. They evaluated the persistence of miRNA alterations after smoking cessation in mice that had been exposed to different doses of CS within 12 h after birth and continued daily for 4 weeks [82]. The two lowest doses (119 and 292 mg/m^3^ of total particulate matter) were not particularly effective, while the highest sublethal dose (438 mg/m^3^) produced extensive miRNA downregulation. Most of the altered miRNAs were restored 1 week after smoking cessation, although some of the mice recovered incompletely. However, when exposed to CS for 4 months, the miRNA changes detected in the mice persisted 3 months after smoking cessation [83]. These results suggest that high doses of and long-lasting exposure to CS are needed to induce irreversible miRNA alterations, which may be involved in CS-related diseases.

CS-induced dysregulation of miRNA has also been demonstrated in humans. Schembri et al. [84] examined whole-genome miRNA expression in bronchial airway epithelium from 10 current smokers and 10 never smokers. Of the 232 detected miRNAs, 28 miRNAs were found to be differentially expressed, with 82% being downregulated in current smokers. Downregulation in smokers compared to never smokers was also observed by Mascaux et al. [85]; they analyzed 60 biopsies of smokers and found that 69 miRNAs were differentially expressed in the smokers and the nonsmokers, the vast majority of which were downregulated. Similar results were detected in human peripheral blood mononuclear cells [86]. Twenty-five miRNAs in human mononuclear cell of peripheral blood were found to be differentially expressed before and after reduced smoke exposure, and most of them were upregulated after smoke reduction, suggesting a trend toward the recovery of miRNAs after smoking cessation. In addition, CS could also induce miRNA changes in human placenta [87] and human spermatozoa [88], which can further alter spermatozoal and/or embryonic gene expression. This finding may partly explain why CS-induced deleterious phenotypes can be transmitted to the progeny of smokers.

CS can also modify circulating miRNAs expression from patients. Takahashi et al. [89] determined the plasma miRNA profiles of 11 smokers and 7 nonsmokers and found that a larger number of miRNAs could be detected in smokers than could be detected in the nonsmokers, and two-thirds of the detected miRNAs (43 miRNAs) were significantly upregulated. Most of the changed miRNAs were previously reported to be potential biomarkers of diseases. Moreover, smoking cessation reversed the altered patterns of expression, which resembled those of the nonsmokers. Another study conducted by Shi et al. [90] also showed dysregulations in circulating miRNAs in smokers compared to the miRNAs in nonsmokers. They investigated the miRNA expression profiles of 28 smokers and 12 nonsmokers and found that 24 miRNAs were upregulated and 11 miRNAs were downregulated in smokers. Interestingly, the dysregulated miRNAs were related to the immune system and hormone regulation. Furthermore, most upregulated miRNAs were associated with hematologic cancers, while most miRNAs upregulated in the plasma of middle-aged smokers were associated with solid cancers. Thus, the CS-changed miRNAs would be potential biomarkers and targets for smoking-associated diseases.

As shown above, the miRNA changes induced by CS are reversible. The miRNA alterations can recover partially or completely when smoking is stopped early. In addition, a number of chemopreventive agents can also partially prevent CS-induced alterations of miRNAs. In a study conducted by Izzotti et al. [91], CS-exposed rats were treated with orally administered the chemopreventive agents *N*-acetylcysteine, oltipraz, indole-3-carbinol, 5,6-benzoflavone, and phenethyl isothiocyanate, as single agents or in combinations. The results showed that none of the above chemopreventive agents appreciably affected the baseline miRNA expression, but all of them attenuated CS-induced miRNA downregulation to a variable extent and in different patterns, indicating the potential safety of these agents and their potential efficacy in preventing miRNA downregulation. The effects of other chemopreventive agents on miRNAs, such as pioglitazone combined with bexarotene [92] and budesonide combined phenethyl isothiocyanate [93], were also observed in the lungs of mice in other studies.

#### Cigarette smoke and lncRNAs

LncRNA has generated considerable interest over the past decade. For a long time, lncRNAs were considered to be nonfunctional, but they are now believed to be involved in various biologic processes, such as oxidative stress, inflammation, cell growth and viability, apoptosis, migration and metabolism at both transcriptional and posttranscriptional levels [94, 95]. The abnormal expression of lncRNAs has been reported to affect the progression of many human diseases, including that of CS-related disorders [96]. Recently, a study of genome-wide lncRNA expression in the lung tissue of nonsmokers (*n* = 5) and smokers with (*n* = 5) or without (*n* = 5) COPD showed that, in smokers without COPD, 87 lncRNAs were significantly upregulated and 244 were downregulated compared to the lncRNAs expressed in the nonsmokers [97]. In the smokers with COPD, 120 lncRNAs were overexpressed and 43 were underexpressed compared with lncRNAs expressed in the smokers without COPD. These results suggest that the differentially expressed lncRNAs induced by CS in the lungs may participate in the pathogenesis and development of COPD and may provide new methods for the diagnosis and treatment of COPD. LncRNA expression profiles in the lung tissue of 5 CS-exposed mice and 5 control mice were also investigated by Zhang and his colleagues [98]. They found that 109 lncRNAs were differentially expressed after CS exposure, a finding that may provide insight useful for determining molecular mechanism of CS-associated oxidative stress and endoplasmic reticulum stress. In smoking-related lung squamous cell carcinoma, 21 lncRNAs were identified, 5 of which were upregulated and 16 of which were downregulated [99]. Further statistical analysis indicated that dysregulation of lncRNA AURKA, BIRC5, and LINC00094 indicated poor prognosis for patient with lung squamous cell carcinoma.

Several in vitro studies showed that CS is able to alter the expression of numerous lncRNAs in airway epithelial cells. Of these lncRNAs, the three most extensively studied with regard to CS are Hox transcript antisense intergenic RNA (*HOTAIR*), colon cancer-associated transcript-1 (*CCAT1*) and metastasis associated in lung adenocarcinoma transcript 1 (*MALAT1*) [100–103]. In CSE-treated HBE cells, the expression of *HOTAIR* and *EZH2* was upregulated [100]. The inhibition of *HOTAIR* and *EZH2* by siRNAs attenuated CSE-induced decreases in *p21* levels, which led to cell cycle aberrations. In addition, in CSE-treated HBE cells, interleukin (*IL*)-6 activated phosphorylated signal transducer and activator of transcription 3 (*STAT3*) and increased the levels of *HOTAIR*, which has been found to be involved in the formation of cancer stem cells and malignant transformation [101]. Similarly, activated *CCAT1* promotes an altered cell cycle transition during the malignant transformation of the HBE cells via epigenetic silencing of *miR*-*218*, suggesting a mechanism for lung cancer development [102]. *MALAT1* increases were also induced by CS, which mediated the epithelial–mesenchymal transition via *EZH2* and enhanced the CSE-induced epithelial–mesenchymal transition and malignant transformation of HBE cells [103]. Other examples that reveal changes in lncRNAs as a consequence of CS include smoke and cancer-associated lncRNA1 (*SCAL1*) [104] and lung cancer progression–association transcript 1 (*LCPAT1*) [105, 106], both of which were upregulated after exposure to CS treatment. Although many studies have investigated the role of lncRNAs in the development of CS-related diseases, the exact mechanism for their effect is not fully understood. The identification of lncRNAs in these processes could provide new clinical targets as well as diagnostic and prognostic tools for CS-related diseases.

## Epigenetics and inflammation

### DNA methylation and inflammation

CS causes multiple inflammatory diseases characterized by the influx of inflammatory cells and secretion of cytokines in susceptible individuals. In recent years, evidence has suggested that epigenetic modification mechanisms can possibly explain the link between CS exposure and inflammation. It has been shown that CS induced hypermethylation of the Runt-related transcription factor 3 (*RUNX3*), Janus kinase 3 (*JAK3*) and keratin 1 (*KRT1*) genes in people deficient for *Alpha*-*1* antitrypsin, which is associated with C-reactive protein (*CRP*) levels [107]. Results from a meta-analysis of epigenome-wide association studies of serum *CRP* and DNA methylation revealed that the methylation at CpG sites that is associated with *CRP* differs by race or ethnicity [108]. Increased DNA methylation with the highest signal, at *cg10636246* near *Absent in melanoma 2* (*AIM2*) and involved in inflammasome response, was associated with lower expression of *AIM2* and lower *CRP* levels. Hypomethylation at *cg18181703* (suppressor of cytokine signaling 3, *SOCS3*), *cg06126421* (tubulin beta, *TUBB*), and *cg05575921* (aryl hydrocarbon receptor repressor, *AHRR*) was associated with higher *CRP* levels and increased risk of future CHD [108]. Moreover, *AHRR* can be hypomethylated by CS [109]. A 2-step epigenetic Mendelian randomization study conducted by Jhun et al. [110] in African Americans suggested that CS may increase serum *IL*-*18* levels through a decrease in the DNA methylation levels of *cg03636183* in the *coagulation factor II (thrombin) receptor*-*like 3* gene. Systemic inflammation has been recognized as a pathogenetic hallmark of inflammatory bowel disease. Researchers found gut segment-specific differences in the DNA methylation profiles of primary intestinal epithelial cells from children with inflammatory bowel diseases versus those from controls, and some of the differentially methylated positions were independent of the mucosal inflammatory activity [111]. Similar results were also published by Somineni et al. [112]. In the blood of 164 pediatric patients with Crohn’s disease taken at diagnosis, they found 1189 CpG sites that were differentially methylated between the patients and controls. Moreover, of the 1189 CpG sites, 1155 (97%) exhibited directional consistency and 872 (73%) showed a statistically significant association with CRP.

Yu et al. [113] confirmed that elevated *DNMT1* was correlated with increased methylation level of the peroxisome proliferator-activated receptor (*PPAR*)-*γ* promoter and decreased expression levels of *PPAR*-*γ*, a nuclear hormone receptor with anti-inflammatory effects, and increased pro-inflammatory cytokine production in peripheral blood monocytes. Moreover, the DNMT inhibitor 5-aza-2′-deoxycytidine (AZA) can promote alternative activation, suppress inflammation by macrophages [114], and inhibit the constitutive activation of pro-inflammatory *NF*-*κB* [115]. Tumor necrosis factor receptor-associated factor 6 (*TRAF6*) is a canonical transduction molecule that plays central roles in activating *NF*-*κB* and MAPK signaling pathways. Feng et al. [116] found that AZA pretreatment significantly enhanced the expression of *IL*-*6* and *IL*-*8* and activated the *NF*-*κB* and MAPK signaling pathways by reducing the methylation levels in the *TRAF6* promoter in human dental pulp cells. In addition to regulating inflammatory factors, *DNMT1* can also be activated by inflammatory factors, such as *IL*-*6*, *transforming growth factor beta 1* [117] and *IL*-*1β* [118].

Tet-eleven translocation proteins (TETs) are dioxygenases that play important roles in decreasing the abundance of DNA methylation. Recently, TETs have gained attention for their roles in the inflammatory process. Upon *Porphyromonas gingivalis* (*Pg*.) lipopolysaccharide (LPS)/interferon (*IFN*)-γ and *Escherichia coli* (*E. coli*) LPS/*IFN*-*γ* stimulation, *TET1* knockdown M1 macrophages exhibited a significant decrease in the production of pro-inflammatory markers such as *IL*-*6* and tumor necrosis factor (*TNF*)-*α* because the *NF*-*κB* signaling pathway was suppressed [119]. Wang et al. [120] found that *TET2* might be a positive regulator of LPS-induced inflammatory response in human dental pulp cells by epigenetically regulating the transcription of the signal transduction molecule *MyD88* in the *NF*-*κB* signaling pathway. However, Zhang et al. [121] showed that *TET2* recruited *HDAC2* and negatively modulated the transcription of *IL*-*6* via histone deacetylation in LPS-activated murine bone marrow-derived dendritic cells and peritoneal macrophages.

These findings indicate that DNA methylation regulates gene expression and may manipulate inflammatory genes to increase or decrease inflammation (Table 2). However, whether the alterations in the DNA methylation profiles are a cause or a consequence of inflammation is still debated. Somineni et al. [112] found that changes in the DNA methylation profiles of blood samples from inflammatory bowel disease patients (at diagnosis) were correlated with acute inflammation. However, with treatment, these changes closely resembled the patterns observed in patients without intestinal inflammation. These results indicate that inflammatory bowel disease-associated patterns of DNA methylation are a result of the inflammatory features of the disease and are less likely to contribute to the development or progression of the disease [112]. In addition, Wahl et al. [122] showed that obesity, an important risk of inflammatory disturbances, can influence DNA methylation. They used genetic association analyses to investigate the potential relationships between BMI (a key measure of adiposity) and DNA methylation in blood and found that the alterations in DNA methylation are predominantly the consequence of adiposity, rather than the cause of it. Therefore, it is possible that CS-induced inflammatory reactions may be a consequence rather than a cause of alterations in DNA methylation patterns. Future research into the identification of heritable epigenetic changes may assist in improving the current understanding of CS-related inflammatory diseases based on the expression of inflammatory markers and the interpretation of their methylation profiles. Understanding the changes in DNA methylation patterns in CS-induced inflammatory diseases may lead to the development of novel therapeutic/management strategies.

**Table 2:** The role of CS-induced aberrant DNA methylation in inflammation

Genes/CpG sites	Changes by CS	Functions on inflammation	Mechanism	Disease	References	Year
*RUNX3*	Hypermethylation [107]	Pro-inflammation	Associated with the level of CRP	COPD	Siedlinski et al. [107]	2012
*JAK3*	Hypermethylation [107]	Pro-inflammation	Associated with the level of CRP	COPD	Siedlinski et al. [107]	2012
*KRT1*	Hypermethylation [107]	Pro-inflammation	Associated with the level of CRP	COPD	Siedlinski et al. [107]	2012
*cg18181703* in *SOCS3*	N/A	Anti-inflammation	Hypomethylation of the CpG site was associated with higher *CRP* levels	CHD	Ligthart et al. [108]	2016
*cg06126421* in *TUBB*	N/A	Anti-inflammation	Hypomethylation of the CpG site was associated with higher *CRP* levels	CHD	Ligthart et al. [108]	2016
*cg05575921* in *AHRR*	Hypomethylation [109]	Anti-inflammation	Hypomethylation of the CpG site was associated with higher *CRP* levels	CHD	Ligthart et al. [108]	2016
cg03636183 in F2RL3	Hypomethylation [110]	Pro-inflammation	Increase IL-18 levels	Hypertension	Jhun et al. [110]	2017
DNMT1	Upregulation [27]	Pro-inflammation	Increase methylation level of the *PPAR*-*γ* promoter and reduce expression of *PPAR*-*γ*	Atherosclerosis	Yu et al. [113]	2016
DNMT1	Upregulation [27]	Pro-inflammation	Regulates IL-6 and TGFβ1; meanwhile, be activated by IL-6 and TGFβ	Benign prostatic hyperplasia	Xu et al. [117]	2017
DNMT1	Upregulation [27]	Pro-inflammation	Be activated by IL-1β via MAPK and NF-κB pathways.	Benign meningiomas	Wang et al. [118]	2016
TET1	N/A	Pro-inflammation	TET1 knockdown reduced IL-6 and TNF-α levels through downregulating NF-κB signaling pathway	Periodontal diseases	Huang et al. [119]	2019
TET2	N/A	Pro-inflammation	Activate the NF-κB signaling pathway	Dental pulp inflammation	Wang et al. [120]	2018

*AML* acute myeloid leukemia, *ARHH* aryl hydrocarbon receptor repressor, *CHD* coronary heart disease, *COPD* chronic obstructive pulmonary disease, *CRP* C-reactive protein, *DNMT* DNA methyltransferase, *F2RL3* coagulation factor II (thrombin) receptor-like 3 gene, *JAK3* Janus kinase 3, *KRT1* keratin 1, *MAPK* mitogen‐activated protein kinase, *NF-κB* nuclear factor kappa B, *PPAR* peroxisome proliferator-activated receptor, *RUNX3* runt-related transcription factor 3, *SOCS3* suppressor of cytokine signaling 3, *TET* Tet-eleven translocation protein, *TGFβ* transforming growth factor beta 1, *TNF-α* tumor necrosis factor α, *TUBB* tubulin beta

### Histone posttranscriptional modifications and inflammation

#### Histone acetylation and inflammation

The acetylation of both histones H3 and H4 has been associated with the promotion of the transcription of pro-inflammatory genes in inflammation. An increase in acetylated histone H4 was found in current smokers, while patients with COPD who had stopped smoking had increased histone H3 acetylation, suggesting that the persistent inflammation in the lungs of the COPD patients after smoking cessation may be regulated by histone H3 acetylation [29]. CS can alter the activity of both HATs and HDACs and thereby enhance histone acetylation, specifically by promoting the expression of *NF*-*κB*-dependent inflammatory genes [29]. In addition to histones, numerous nonhistone proteins can also be acetylated and deacetylated by HATs and HDACs, respectively [123]. Studies have shown that *NF*-*κB* can be acetylated at multiple lysine residues. The acetylation of the *NF*-*κB p65* subunit at lysine 310 or lysine 221 increases the transcriptional activity or DNA-binding affinity of *NF*-*κB*, whereas acetylation at lysine 122 and lysine 123 facilitates the relocation of *NF*-*κB* from the nucleus [124]. Therefore, CS can promote inflammation by acetylating *NF*-*κB* by upregulating HATs and inhibiting HDACs.

Evidence is emerging that HATs/HDACs have important roles in the inflammatory response and that HDAC inhibitors may have clinical application in the treatment of inflammatory disease [125]. *CBP*/*p300* is the most extensively studied HAT. Increased acetylation of histone (H3 and H4) and *NF*-*κB* by *CBP*/*p300* is associated with CS-mediated pro-inflammatory cytokine release, which is responsible for the sustained pro-inflammatory response observed in COPD [126]. CS increased the expression of pro-inflammatory mediators *IL*-*8*, *TNF*-*α* and *matrix metalloproteinase 9* through *HDAC1* depression in rat lungs and in macrophages, while knocking down *HDAC1* increases the expression of these genes [37]. In smokers with COPD, the levels and activities of *HDAC2* are significantly reduced, leading to increased airway inflammation by promoting the expression *of IL*-*17A* [127] and/or the phosphorylation of *Akt* [128] and/or the activity *of NF*-*κB* [129]. Barnes et al. [130] concluded that decreased *HDAC2* accounts for the amplified inflammation and resistance to the actions of corticosteroids. Furthermore, *HDAC2*-deficient mice are not responsive to budesonide, which inhibits LPS-induced lung inflammation [131]. Moreover, corticosteroids can suppress inflammation by recruiting *HDAC2* at *NF*-*κB*-driven pro-inflammatory gene promoters, thereby inhibiting the transcription of these genes [132]. Enhanced HDAC activity by low-dose theophylline treatment can improve steroid responsiveness and reduce airway inflammation [133]. Thus, recruitment of *HDAC2* to activated inflammatory genes is a major mechanism of inflammatory gene repression by corticosteroids. *HDAC3* has been reported to be an important player in the recruitment of monocytes to sites of inflammation and in macrophage cytokine production. Acute smoke exposure has been shown to reduce *HDAC3* activity in human alveolar macrophages, where it resulted in increased production of *IL*-*8* and *IL*-*1β* [40]. *HDAC4* functions as a corepressor of multiple immunologically related transcription factors, including *c*-*Jun* and *IL*-*17A*, and downregulates *IL*-*6* expression [134]. Both Poralla et al. [135] and Zhao et al. [136] confirmed a pro-inflammatory capacity of *HDAC5* in macrophages by activating *NF*-*κB* signaling pathway. Thus, *HDAC5* overexpression promotes the production of inflammatory cytokine, and *HDAC5* depletion inhibits the production of inflammatory cytokine. Studies on *HDAC6* that is activated by CS [42] have shown a positive relationship between *HDAC6* and inflammatory response. It has been shown that *HDAC6* inhibition confers protective effects against LPS-induced inflammation, as is demonstrated by the reduced production of the pro-inflammatory cytokines *TNF*-*α*, *IL*-*1β*, and *IL*-*6* and decreased leukocyte infiltration [137, 138]. Zhang et al. [139] found that You-Gui pills (You-Gui-Wan; YGW) reduced the production of inflammatory cytokines, such as *IL*-*4*, *IL*-*5*, and *IL*-*13*, by increasing the corresponding activity of *HDAC7*, *HDAC710*, *HDAC11*, and *HDAC9*–*11*, suggesting that *HDAC7*- and *HDAC9*–*11*-induced histone deacetylation of memory T lymphocytes has a protective role against lung inflammation and eosinophilic infiltration. *HDAC8* has been shown to attenuate acetylation of Histone H3 and H4 in the *IFN*-*β* promoter, resulting in suppression of *IFN*-*β* transcription, which leads to the inhibition of innate *IFN*-*β* production [140]. Another study shows the opposite result: specific inhibition of *HDAC8* reduces LPS-induced *IL*-*1β*, *TNF*-*α*, *IL*-*6* secretion [141]. Evidence is emerging that inhibiting HDAC activity by HDAC inhibitors has clinical applications for anti-inflammatory treatment because of the suppressive effects of these inhibitors on pro-inflammatory molecules; that is, they can reduce the *NF*-*κB*-dependent production of pro-inflammatory cytokines, including *IL*-*2*, *TNF*-*α IL*-*6*, *IL*-*1β*, and *IFN*-*γ*, under various pathological conditions, which are triggered by respiratory syncytial virus, mitogenic anti-CD3, LPS, and phorbol myristate acetate [142]. Multiple HDACs are involved in inflammation. In some cases, more than two HDACs play opposite roles in regulating the inflammatory response, suggesting that pan-HDAC inhibitors may result in unwanted effects. Therefore, the detailed mechanism underlying the HDAC regulation of cytokine production and secretion need to be further investigated.

SIRTs, class III HDACs, have been identified as important regulators of the inflammatory process over the past few years. In *TNF*-*α*-stimulated human keratinocytes, the expression levels of *SIRT1*, *SIRT2*, *SIRT3*, *SIRT4* and *SIRT5* are downregulated, while those of *SIRT6* and *SIRT7* are upregulated, indicating a necessary function of SIRTs in inflammation [143]. Both *SIRT1* and *SIRT2* are reported to deacetylate the *RelA*/*p65* subunit of *NF*-*κβ* at lysine 310, inhibiting *NF*-*κβ* signaling, while *SIRT6* has been shown to interact with the *p65*/*RelA* that is bound to the *NF*-*κβ* promoter region to repress transcriptional activity, which results in reduced expression levels of inflammatory factors [144]. Further studies have shown that knocking down *SIRT1*, *2* or *6* both in vivo and in vitro results in increased expression of *NF*-*κβ* and simultaneous overexpression of inflammatory cytokines [145–147]. *SIRT3* may serve as an important target that regulates inflammation. SIRT3 overexpression could ameliorate the activation of Nod-like receptor protein 3 (*NLRP3*) inflammasome, while *SIRT3* knockdown accelerates the induction of *NLRP3* expression [148]. It has been shown that *SIRT3* can suppress inflammation by decreasing the expression levels of *NF*-*kB*, high mobility group box 1 (*HMGB1*), *c*-*Jun*, *c*-*Fos*, cyclooxygenase-2 (*COX2*), *TNF*-*α*, *IL*-*1β* and *IL*-*6* [149]. *SIRT4* is also found to negatively regulate CSE-induced *NF*-*κB* activation via inhibiting the degradation of inhibitory kappa B (*IκB*) kinase α [47]. The pro-inflammatory cytokines *IL*-*1β*, *TNF*-*α*, and *IL*-*6*, the downstream target genes of *NF*-*κB*, are inhibited by overexpression of *SIRT4* [47]. Vakhrusheva et al. [150] showed that the absence of *SIRT7* produces an increase in inflammation, illustrating that *SIRT7* also functions to suppress inflammation. In contrast to other SIRTs, *SIRT5* has a role in promoting inflammation. Qin et al. [151] showed that *SIRT5* competes with *SIRT2* to interact with *NF*-*κB p65*, in a deacetylase activity-independent manner to block the deacetylation of *p65* by *SIRT2*, which leads to the increased acetylation of *p65* and the activation of the *NF*-*κB* pathway and its downstream cytokines. In recent years, there have been an increasing number of studies with accumulating evidence confirming the importance of histone acetylation state in inflammation (Table 3). Targeting histone acetylation might be a new means of therapeutically targeting inflammatory processes.

**Table 3:** Studies reporting aberrant HATs/HDACs induced by CS in inflammation

HATs/HDACs	Changes by CS	Functions on inflammation	Mechanism	Disease	References	Year
CBP/p300	Upregulation [50]	Pro-inflammation	Increase acetylation of histones (H3/H4) and NF-κB	COPD	Rajendrasozhan et al. [126]	2009
HDAC1	Downregulation [37]	Anti-inflammation	Decrease level of acetylated H3K9	COPD	Chen et al. [37]	2015
HDAC2	Downregulation [29, 30, 38]	Anti-inflammation	Inhibit IL-17A Suppress the phosphorylation of Akt Inhibit NF-κB	COPD Asthma COPD	Lai et al. [127] Xia et al. [128] To et al. [129]	2018 2018 2017
HDAC3	Downregulation [39, 40]	Anti-inflammation	Repress synthesis of NF-κB -driven inflammatory cytokine	COPD	Winkler et al. [40]	2012
HDAC4	Downregulation [38]	Anti-inflammation	Repress c-Jun and IL-17A	COPD	Lu et al. [134]	2015
HDAC5	Downregulation [29, 30]	Pro-inflammation	Activate NF-κB	Chronic inflammatory diseases Mycoplasma pneumoniae pneumonia	Poralla et al. [135] Zhao et al. [136]	2015 2019
HDAC6	Upregulation [41] Activation [42]	Pro-inflammation	Promote the increase of matrix metalloproteinase 9 expression and activate NF-κB by inducing IĸB phosphorylation Promote the phosphorylation of p38 MAPK	Acute lung injury Brain inflammation	Liu et al. [137] Song et al. [138]	2019 2019
HDAC7	Downregulation [35]	Anti-inflammation	Increase histone deacetylation of memory T lymphocytes	Asthma	Zhang et al. [139]	2015
HDAC8	Downregulation [29, 30]	Anti-inflammation Pro-inflammation	Promote the acetylation modification of IFNβ1 promoter, thus selectively increasing innate IFN-β production Increase the production of IL-1β, TNFα, and IL-6	Asthma Systemic juvenile idiopathic arthritis	Meng et al. [140] Li et al. [141]	2016 2015
HDAC9	N/A	Anti-inflammation	Increase histone deacetylation of memory T lymphocytes	Asthma	Zhang et al. [139]	2015
HDAC10	Downregulation [36]	Anti-inflammation	Increase histone deacetylation of memory T lymphocytes	Asthma	Zhang et al. [139]	2015
HDAC11	N/A	Anti-inflammation	Increase histone deacetylation of memory T lymphocytes	Asthma	Zhang et al. [139]	2015
SIRT1	Downregulation [43]	Anti-inflammation	Deacetylate the RelA/p65 subunit of NF-κβ and attenuate NF-κB-mediated gene transcription	Chronic inflammatory diseases	Schug et al. [145]	2010
SIRT2	N/A	Anti-inflammation	Deacetylate the RelA/p65 subunit of NF-κB at Lys310 and inhibit NF-κβ signaling	Inflammatory bowel disease	Lo et al. [146]	2014
SIRT3	Downregulation [46]	Anti-inflammation	Ameliorate NLRP3 inflammasome activation Decrease the expression levels of NF-κB, HMGB1, c-Jun, c-Fos, COX2, TNF-α, IL-1β and IL-6	Hyperlipidaemia Chronic kidney disease	Liu et al. [148] Qiao et al. [149]	2018 2018
SIRT4	Downregulation [47]	Anti-inflammation	Suppress NF-κB activating via inhibiting the degradation of IκBα	COPD	Chen et al. [47]	2014
SIRT5	Downregulation [48]	Pro-inflammation	Compete with SIRT2 to interact with NF-κB p65 to block the deacetylation of p65 by SIRT2, leading to increased acetylation of p65 and the activation of NF-κB pathway	Sepsis	Qin et al. [151]	2017
SIRT6	Downregulation [49]	Anti-inflammation	Interact with p65/RelA bound to the NF-κβ promoter region and repress transcriptional activity	Inflammatory vascular diseases	Lappas M. [147]	2012
SIRT7	N/A	Anti-inflammation	Deacetylate p53, leading to inactivation of p53	Heart diseases	Vakhrusheva et al. [150]	2008

*CBP* cAMP-response element binding protein, *COX2* cyclooxygenase-2, *CS* cigarette smoke, *HAT* histone acetyltransferases, *HDACs* histone deacetylases, *HMGB1* high mobility group box 1, *IFNβ1* interferon beta 1, *IκBα* inhibitory kappa B kinase α, *IL* interleukin, *NLRP3* NOD-like receptor family, pyrin domain containing 3, *SIRT* sirtuin

#### Histone methylation and inflammation

HMTs have been proven to have significant regulatory effects on inflammatory responses. *EZH2*, the primary H3K27 tri-methyltransferase, was found to integrate the complex functions of *TNF*-*α* signaling to enhance the inflammatory response. *EZH2* deficiency directly stimulates *TRAF* expression to enhance *TNF*-*α*-induced *NF*-*κB* signaling, thereby leading to uncontrolled inflammation [152]. Liang et al. [153] found that *EZH2* can induce caspase-3 activation and production of pro-inflammatory molecules by activating *p38*. This effect can be reversed by inhibiting *EZH2*. Recently, PRMTs have been confirmed to be correlated with inflammatory responses. *PRMT1*, *PRMT4*, *PRMT5* and *PRMT6* are all reportedly involved in inflammatory responses in association with the pro-inflammatory factor *NF*-*κB*, although each of those has unique regulatory mechanisms [154]. In addition, elevated *PRMT1*, stimulated by *IL*-*4*, plays a crucial role in chronic antigen-induced pulmonary inflammation [155]. In LPS-induced macrophages, the dose of *PRMT2* influences the lung airway hyper-responsiveness, the recruitment of neutrophils, and the expression of pro-inflammatory cytokines, such as *IL*-*6* and *TNF*-*α*, through *NF*-*κB* [156]. *PRMT5* was shown to bind and methylate *HOXA9* in *TNF*-*α*-stimulated endothelial cells to regulate inflammatory processes of these cells [157]. The inhibition of *PRMT5* by *PRMT5* inhibitor or by siRNA-mediated knockdown prevents the phosphorylation of *IκBβ* and *IκBα* and inhibits the nucleus translocation of *p65*, leading to a reduction in *IL*-*6* and *IL*-*8* expression [158]. Our previous mouse model and in vitro studies revealed that *PRMT6* exerts a negative effect on CS-induced pulmonary inflammation through H3R2me2a, while overexpression of *PRMT6* inhibits CS-induced inflammation [51, 52]. Thus, HMTs play important roles in the process of inflammatory gene posttranslational modification. Given the importance of inflammation in a great number of diseases, HMTs appear to be a newly discovered potential therapeutic target to modulate the inflammatory response. However, current studies linking HMTs to inflammation are in a very nascent stage. Future studies are warranted to address the underlying molecular mechanism of histone methylation and HMTs in the regulation of inflammatory gene transcription.

#### Histone phosphorylation and inflammation

Previous studies indicated that the phosphorylation of histones is responsible for inflammatory processes [159]. *COX2* is an important enzyme in activating the cellular inflammatory response. It has been shown that the phosphorylation of histone H3 at Ser10 and Ser28 plays a critical role in promoting *COX2* expression [159]. Histone H3 phosphorylation at Ser10, which occurs at the promoters of the *NF*-*κB*-regulated cytokines, has been observed to increase the accessibility of *NF*-*κB* binding sides [160]. Chung et al. [161] find that the activation of *IκBα* is critical in the phospho-acetylation of histone H3 on the pro-inflammatory gene promoters in response to CS stimuli and the histone H3 phospho-acetylation is critical for the activation of *NF*-*κB*-directed gene expression. It has been shown that *MSK1* can induce *p65* phosphorylation at Ser276, which in turn induces *IL*-*8*/*CXCL8* release in thrombin-stimulated human lung epithelial cells [162].

Chemokine fractalkine (*CX3CL1*) is essential in neuroinflammation. Galán-Ganga et al. [163] found that knocking down of *MSK1* decreased the mRNA levels of *IL*-*1β*, *TNF*-*α*, which was triggered by stimulation with *CX3CL1* through the inactivation of *NF*-*κB* signaling pathway. In addition, *MSK1* was also shown to be an important downstream kinase involved in CS-induced *NF*-*κB* activation and the phospho-acetylation of histone H3 [57]. The *NF*-*κB* inhibitor dimethyl fumarate can inhibit *NF*-*κB*-dependent chemokine secretion by inhibiting histone H3 phosphorylation through *MSK1* [160]. The *IL*-*1* receptor antagonist (*IL*-*1Ra*) is homologous to *IL*-*1* and is able to bind to, but not activate, the *IL*-*1* receptor. It has been shown that *MSK1* and *MSK2* are required for the induced expression of both *IL*-*1Ra* mRNA and protein, while knocking out MSK in mice was found to result in a decreased *IL*-*1Ra* production [164]. These results indicate a promoting effect of *MSK1* on inflammation. However, Zhong et al. [165] put forward a different opinion, suggesting that *MSK1* is negatively correlated with astrocyte inflammation and can inhibit inflammatory cytokine production. They showed that *MSK1* inhibition promoted the expression of *TNF*-*α*, *IL*-*6*, *IL*-*10*, and *IL*-*1β*. Thus, *MSK1* might either promote or repress inflammation depending on the cell type or stimuli. RSK is another kind of kinase that catalyzes the phosphorylation of histone H3. Lin and his colleagues [166] found that the thrombin-induced release and activation of the inflammatory factor *IL*-*8*/*CXCL8* can be inhibited by *RSK1* siRNA, suggesting that *RSK1* has an enhancing effect in the inflammatory process. Therefore, CS may induce inflammatory responses through the histone phosphorylation induced by activated kinases; these kinases may represent potential targets in therapy that controls the CS-mediated chronic inflammatory response.

#### Histone ubiquitination and inflammation

Over the last few years, evidence has accumulated showing that ubiquitination is a key process in regulating inflammation responses [167]. Studies in vivo and in vitro have revealed the involvement of the ubiquitin proteasome system in CS-induced inflammation. The muscle-specific E3 ubiquitin ligases *MuRF1* and *MAFbx*/*atrogin*-*1*, which play important roles in the process of muscle-wasting degradation, were shown to be upregulated by CS [73]. This process is related to *IκB*-*α* degradation and *NF*-*κB* activation. *NF*-*κB* inhibition prevents CS-induced upregulation of *MuRF1* and CS-induced reduction in the diameter and degradation of the *MyHC*-related C2 myotubes [2], suggesting that inflammation is responsible for CS-induced proteasomal degradation in skeletal muscle. *Atrogin*-*1* is a ubiquitin protein ligase. Zeng et al. [168] found that *atrogin*-*1* overexpression markedly inhibits the expression of pro-inflammatory-related genes (including *IL*-*1β*, *IL*-*6*, *Ptgs2* and *Serpinb2*), while knocking down atrogin-1 by siRNA had the opposite effects. *MUL1*, an *Akt* ubiquitin E3 ligase that catalyze *Akt* ubiquitination/degradation, can be upregulated by CS [75]. Studies have shown that the knocking down *MUL1* with a combination of siRNA and shRNA results in potentiated activation of both the *IFN*-*β* and *NF*-*kB* pathways [169]. *HDAC2*, which can suppress the inflammatory genes via deacetylation of histones and glucocorticoid receptors [130], was ubiquitinated and degraded in response to CS and oxidative stress [77]. It has been shown that, in CSE-exposed airway epithelial cells and macrophages, *HDAC2* is excessively ubiquitinated and degraded in the proteasome due to the low expression of *USP17*, an important deubiquitinating enzyme [77]. Overexpressed *USP17* blocks the degradation of *HDAC2* induced by CSE, resulting in increased production of inflammatory factors. The *NLRP3* inflammasome consists of a protein-nucleotide-binding domain and leucine-rich repeat pyrin-containing protein-3, an apoptosis-associated speck-like protein containing *CARD* and the pro-apoptotic protease caspase-1. Upon inflammasome activation, caspase-1 is cleaved and activated, and pro-inflammatory cytokines such as *IL*-*1β* and *IL*-*18* are released [170]. Han et al. [171] reported that CSE can induce the degradation of *NLRP3* protein via the ubiquitin proteasome system in human monocytes and macrophages, which in turn diminishes the formation of *NLRP3* inflammasomes and prevents the subsequent release of *IL*-*1β* and *IL*-*18*. However, the levels of *IL*-*1β* and *IL*-*18* were increased in the lungs, lavage fluid, and sputum of the COPD subjects and animals exposed to CS [172, 173]. It is possible that the response to CS could differ by cell type, model system, or kinetics [171]. Pulmonary cells such as lung epithelial cells or alveolar macrophages could have different responses to CSE in terms of cellular *NLRP3* protein levels, while monocytes or monocyte-derived macrophages have decreased *NLRP3* protein levels upon exposure to CS, leading to immunosuppression [171]. These results show that CS can elevate the level of ubiquitination through the upregulation of E3 ubiquitin ligases or the decrease of deubiquitinating enzymes, resulting in decreased inflammatory responses and immunosuppression. Thus, CS may suppress immune function by modifying the activity of the ubiquitin proteasome system, making smokers more susceptible to infection.

### Non-coding RNAs and inflammation

#### MiRNAs and inflammation

In the last few years, there has been a growing interest in the role of miRNAs in the CS-related inflammatory process. MiRNAs have distinctive functions depending on their expression in different cell types, and they can directly or indirectly regulate the inflammatory response. Additionally, some proteins that are induced during inflammatory responses can regulate the processing of miRNAs [174]. The role of a few selected miRNAs changed by CS in inflammation is discussed below (Table 4). It is noteworthy that several miRNAs dysregulated due to CS, namely, including *let*-*7*, *miR*-*16*, *miR*-*21*, *miR*-*24*, *miR*-*29b*, *miR*-*30*, *miR*-*132*, *miR*-*135b*, *miR*-*145*, *miR*-*146a*, *miR*-*149*, *miR*-*150*, *miR*-*181*, *miR*-*195*, *miR*-*200*, *miR*-*212*, *miR*-*218*, *miR*-*223*, and *miR*-*320* are found to be functionally related to inflammation. Among these, *miR*-*132* is upregulated in the serum of smokers compared with its expression in the nonsmoker controls, and the *miR*-*132* expression level was positively correlated with the levels of inflammatory cytokines (*IL*-*1β* and *TNF*-*α*). Knocking down *miR*-*132* attenuates CS-induced inflammation in human monocyte-like cells by targeting the *SOCS5*, which acts to limit the duration of signaling responses [175]. *MiR*-*195* was highly expressed in peripheral lung tissues from COPD patients compared to the levels found in never smokers. Knocking down *miR*-*195* alleviates CS-induced lung injury and inflammatory cell infiltration, as well as production of *TNF*-*α* and *IL*-*6*, by regulating *Akt* signaling [176], indicating that *miR*-*195* has a role in the promotion of inflammation. However, there are still disputes about the effects of miR-195 on inflammation. *MiR*-*195* expression is decreased significantly in the serum samples of ulcerative colitis patients compared to the levels in normal subjects. In addition, the expression of *miR*-*195* is further decreased in steroid-resistant ulcerative colitis patients compared with its expression in steroid-sensitive ulcerative colitis patients. Upregulating *miR*-*195* represses *Smad7* gene expression, which can aggravate inflammation through the inhibition of the TNF-β signal pathway [177]. These results indicate that the decrease in *miR*-*195* might act in the steroid resistance of ulcerative colitis by activating inflammation through *Smad7* signaling.

**Table 4:** Implications of CS-related microRNAs on inflammation

miRNAs	Changes by CS	Functions on inflammation	Mechanism	Disease	References	Year
Let-7c	Downregulation [80, 82]	Anti-inflammation	Suppress NF-κB signaling Suppress STAT3 signaling	Endometritis COPD	Zhao et al. [181] Yu et al. [182]	2019 2016
miR-16	Upregulation [196]	Pro-inflammation	Upregulated by NF-κB	Gastric cancer	Shin et al. [196]	
miR-21	Upregulation [195, 196]	Pro-inflammation	Upregulated by NF-κB	Gastric cancer	Shin et al. [196]	
miR-24	Downregulation [194]	Anti-inflammation	Suppress TNF-α, IL-1β and NF-κB	IPF	Ebrahimpour et al. [194]	2019
miR-29b	Downregulation [195]	Anti-inflammation	Reduce VEGF-A expression; meanwhile upregulated by NF-κB inhibitor	Breast cancer	Malik et al. [195]	2018
miR-30	Downregulation [80, 82]	Anti-inflammation	Inhibit NF-κB	N/A	Izzotti et al. [80]	2009
miR-132	Downregulation [175]	Anti-inflammation	Repress SOCS5 expression	COPD	Diao et al. [175]	2018
miR-135b	Upregulation [183]	Anti-inflammation	Upregulated by IL-1R1 and directly targets IL-1R1 in a negative regulatory feedback loop	N/A	Halappanavar et al. [183]	2013
miR-145	Downregulation [187]	Anti-inflammation	Suppress Kruppel-like 5 and NF-κB	COPD	Dang et al. [187]	2019
miR-146a	Downregulation [80]	Anti-inflammation	Reduce CS-induced COX-2 production.	N/A	Zago et al. [180]	
miR-149	Downregulation [188]	Anti-inflammation	Inhibit the TLR-4/NF-κB signaling pathway	COPD	Shen et al. [188]	2017
miR-150	Downregulation [189]	Anti-inflammation	Inactivate NF-κB	COPD	Xue et al. [189]	2018
miR-181	Downregulation [190]	Anti-inflammation	Inhibit CCN1	COPD	Du et al. [190]	2017
miR-195	Upregulation [176]	Pro-inflammation Anti-inflammation	Elevate Akt phosphorylation by suppressing PHLPP2 expression Inhibit NF-κB and JNK signaling by repressing Smad7	COPD Ulcerative colitis	Gu et al. [176] Chen et al*. [*177*]*	2018 2015
miR-200	Downregulation [197]	Anti-inflammation	Suppressed by NF-κB	Lung cancer	Zhao et al. [197]	2013
miR-212	Downregulation [191]	Anti-inflammation	Promote the phosphorylation of Akt.	COPD	Jia et al. [191]	2018
miR-218	Downregulation [192]	Anti-inflammation	Inactivate NF-κB	COPD	Conickx et al. [192]	2017
miR-223	Downregulation [80] Upregulation [186]	Anti-inflammation Pro-inflammation	Inhibit NF-κB by targeting IL-1 receptor-associated kinase 1 Downregulate NLRP3 Suppress HDAC2	Low back pain Inflammatory bowel disease COPD	Wang et al. [184] Neudecker et al. [185] Leuenberger et al. [186]	2018 2017 2016
miR-320	N/A	Anti-inflammation	Inhibit NF-κB	COPD	Faiz et al. [198]	2019

*CCN1* a member of CCN family, *IPF* idiopathic pulmonary fibrosis, *PHLPP2* PH domain and leucine-rich repeat protein phosphatase 2, *STAT3* signal transducer and activator of transcription 3, *TLR* toll-like receptor, *VEGF* vascular endothelial growth factor. *JNK* c-Jun N-terminal kinase

*MiR*-*146a* was originally identified as an inflammatory response miRNA that was known to target members of the *NF*-*κB* family [178]. The expression level of *miR*-*146a* was shown to be decreased after CS exposure [80]. *RelB*, an *NF*-*κB* protein, exhibited potent anti-inflammatory properties against CS by reducing the production of *COX2* [179]. It has been shown that *RelB* inhibits CS-induced *COX2* expression by upregulating *miR*-*146a*, which highlights the potential of the *RelB*-*miR*-*146a* axis as a novel regulatory pathway that can attenuate inflammation in response to CS [180]. *Let*-*7c* was previously reported to be downregulated in rat and mouse lungs upon CS exposure [80, 82]. It has been shown that *let*-*7c* can suppress LPS-induced inflammation by inhibiting *NF*-*κB* [181] and *STAT3* [182]. Thus, *let*-*7c* may function as a protective effector by conferring protection against CS-induced inflammation. *MiR*-*30* is downregulated remarkably, even at the lowest dose of CS exposure [80, 82]. This miRNA is involved in *NF*-*κB* activation, which represents a general defense mechanism against stress and toxic agents [80]. *MiR*-*135b* was found to be significantly upregulated in the lungs of CS-exposed mice and in lung epithelial cells following *IL*-*1 receptor*-*1* stimulation [183]. M*iR*-*135b* can also bind to its own regulator (*IL*-*1 receptor*-*1*), as well as its downstream effector, Caspase-1, in an attempt to stop further expansion of the inflammatory process by other potent cytokines, such as *IL*-*1β*, implying that these molecules self-regulate in a negative feedback loop [183]. *MiR*-*223* was shown to be downregulated in the lungs of rats exposed to CS [80]. It has been shown that the overexpression of *miR*-*223* suppresses LPS-induced *NF*-*κB* signaling by targeting *IL*-*1* receptor-associated kinase 1 [184]. Recent data have revealed the upregulation of *miR*-*223* as a novel biomarker in subsets of patients with inflammatory bowel disease [185]. Mice genetically deficient in *miR*-*223* displayed markedly exacerbated experimentally induced colitis, hyperactivated *NLRP3*, and *IL*-*1β* release, while mice with overexpressed miR-223 displayed the opposite effects [185], suggesting that *miR*-*223* represents a novel target for reducing the inflammatory response. In contrast, Leuenberger et al. [186] found elevated expression levels of *miR*-*223* in COPD patients and in CS-exposed mice. The increased expression of *miR*-*223* repressed the expression and activity of *HDAC2* in pulmonary cells, which, in turn, alter the expression profile of chemokines.

The levels of *miR*-*145* [187], *miR*-*149* [188], *miR*-*150* [189], *miR*-*181* [190], *miR*-*212* [191] and *miR*-*218* [192] were decreased significantly in vivo and in vitro during exposure to CS, and the expression of inflammatory genes was simultaneously increased. Overexpression of *miR*-*145*, *miR*-*149*, *miR*-*150* or *miR*-*218* suppressed the secretion of inflammatory cytokines that had been induced by CS through the inactivation of the *NF*-*κB* signaling pathway [187–189, 192]. *miR*-*212* reduced inflammation by promoting the phosphorylation of *Akt* [191], while *miR*-*181* decreased the inflammatory response, neutrophil infiltration, and inflammatory cytokine production by targeting *CCN1* [190]. *CCN1*, also known as *Cyr61*, was found to be upregulated in lung epithelial cells by CSE via the induction of ROS and endoplasmic reticulum stress, which subsequently resulted in augmented *IL*-*8* release through the activation of the *Wnt* pathway [193]. Nicotine reduced *miR*-*24* expression in a dose-dependent manner and upregulated the expression of inflammatory cytokines such as *TNF*-*α*, *IL*-*1β* and *NF*-*κB* in primary human lung epithelial cells [194]. In addition, the induced expression of these inflammatory molecules by nicotine was mirrored by genetic ablation of *miR*-*24*, suggesting that nicotine controls these molecules, at least in part, through the inhibition of *miR*-*24* [194].

Benzo[a]pyrene, a constituent of CS, has been shown to promote inflammation pathways via *TNF*-*α* and *NF*-*κB*, leading to *IL*-*6* upregulation, miRNA (*let*-*7a*, *miR*-*21* and *miR*-*29b*) dysregulation and the activation of *VEGF* [195]. The downregulation of *let*-*7a* and *miR*-*29b* and the upregulation of *miR*-*21* induced by benzo[a]pyrene in human mammary cells were associated with the altered expression of inflammation mediators. Moreover, pretreatment with an *NF*-*κB* inhibitor attenuated the benzo[a]pyrene-mediated dysregulation of the miRNAs. In addition, Shin et al. [196] found that the upregulation of *miR*-*16* and *miR*-*21* induced by nicotine stimulation can be restored by inhibiting *NF*-*κB*. Zhao et al. [197] showed that CS induced the activation of *NF*-*κB* and downregulated the expression of *miR*-*200c*. The inhibition of *NF*-*κB* activation also reversed the CS-induced decrease in *miR*-*200c* expression. These results suggest that *let*-*7a*, *miR*-*16*, *miR*-*21*, *miR*-*29* and *miR*-*200c* are the downstream targets of *NF*-*κB* that are involved in *NF*-*κB*-mediated inflammation. *CXCL8* is a key mediator in the neutrophil infiltration into airway tissue by acting as a neutrophil chemoattractant. It has been observed that *miR*-*320d* suppressed the CSE-induced release of *CXCL8* via the inhibition of *NF*-*κB* activation, thus exhibiting its anti-inflammatory function [198]. Moreover, inhaled corticosteroids can induce increase in *miR*-*320d*. These studies open a window for certain miRNAs to serve as diagnostic and therapeutic agents against CS-induced inflammation.

#### LncRNAs and inflammation

In recent years, lncRNAs are emerging as biomarkers with diagnosis value in prognosis protocols or in the personalized treatment of inflammation-related alterations. Several reports have demonstrated the dysregulation of lncRNAs in the pathogenesis of various inflammatory diseases [199]. The abnormal expression of lncRNA in inflammatory diseases is related to the expression of many inflammatory genes and the activation or suppression of signaling pathways. However, until now, little has been known about lncRNAs in the context of CS-related inflammation. As mentioned above, the lncRNA expression profiles are changed when exposed to CS, and *HOTAIR*, *MALAT1*, and *CCAT1* have been the most extensively studied of these lncRNAs. It has been reported that *HOTAIR* may be considered a promoter of inflammatory response [200]. In a sepsis mice model, LPS can induce the upregulation of *HOTAIR* in cardiomyocytes, in line with increased *NF*-*κB* activation and *TNF*-*α* production, which is reversed by *HOTAIR* silence [201]. Furthermore, *HOTAIR*-induced *TNF*-*α* production is suppressed by an *NF*-*κB* inhibitor, suggesting that *HOTAIR* can promote *TNF*-*α* production by activating the *NF*-*κB* signaling pathway [201]. Protein Kinase Resource (*PKR*) has been identified as a necessary protein kinase for the activation of one or several types of inflammasomes [202]. Ultraviolet B rays can promote the expression of *HOTAIR* in human keratinocytes, which in turn promotes the expression of *PKR*, *TNF*-*α* and *IL*-*6* [203]. Suppressing *HOTAIR* decreases the expression of *PKR* as well as *TNF*-*α* and *IL*-*6*. A similar result was also found by Liu et al. [204]; that is, *IL‐6*, *IL‐1β*, *COX2*, and *TNF*-*α* protein levels are significantly depressed by silencing *HOTAIR* but are increased by upregulation of *HOTAIR*. Recently, lncRNA *MALAT1* was also identified as a regulator of inflammatory cytokine production. *MALAT1* can work as a novel inflammatory regulator that acts through the *p38 MAPK*/*NF*-*κB* pathway [205]. In the cecal ligation and puncture (CLP)-induced sepsis model, *MALAT1* expression is significantly upregulated, which in turn aggravates cardiac dysfunction and inflammation by activating *p38 MAPK*/*NF*-*κB*, resulting in increased *TNF*-*α*, *IL*-*1β*, *IL*-*6*, *IL*-*10*, *IL*-*17* and *IFN*-*γ*. In addition, these effects induced by CLP are reversed by knocking down *MALAT1* [205]. A recent study also found that, under hyperglycemic conditions, *MALAT1* expression is increased significantly in human umbilical vein endothelial cells, which is associated with a parallel increase in serum amyloid antigen 3, an inflammatory ligand and target of *MALAT1* [206]. Knocking down *MALAT1* downregulates serum amyloid antigen 3 activation, subsequently reducing the RNA and protein expressions of key inflammatory mediators (*IL*-*6* and *TNF*-*α*) [206]. LncRNA *MALAT1* was found to interact with *NF*-*κB* in the nucleus, thus inhibiting the DNA-binding activity of *NF*-*κB* and reducing the production of inflammatory cytokines. Knocking down *MALAT1* can increase LPS-induced expression of *TNF*-*α* and *IL*-*6* [207]. In addition to regulating the expression of inflammatory factors, both HOTAIR and *MALAT1* can be upregulated by the pro-inflammatory cytokine *IL*-*6* [101, 208]. Moreover, blocking *IL*-*6* with an anti-*IL*-*6* antibody significantly reduces *MALAT1* expression [208]. These results suggest that a feedback regulation may involve *IL*-*6*, *HOTAIR* and *MALAT1. CCAT1* is highly expressed in inflammatory bowel disease patients and in colorectal cancer cells. It has recently been shown that *CCAT1* triggers *TNF*-*α* and *IFN*-*γ* expression by targeting *miR*-*185* in human colorectal cancer cells. In addition, the expression of *CCAT1* is significantly increased after *TNF*-*α* treatment, forming a positive regulatory feedback loop [209]. In summary, lncRNAs play roles in the regulation of gene transcription during the inflammatory response and are closely related to CS-induced inflammatory diseases (Table 5). Because of the complex pathogenesis of CS-induced inflammation and the impact of many factors, the function of many lncRNA is still not clear. Thus, substantial further researches are needed to illuminate the relationship between lncRNA and CS-induced inflammation. With the increased in-depth research on lncRNA function, it is expected to become a new target for the diagnosis of CS-related inflammatory diseases and a new therapeutic target.

**Table 5:** CS‐regulated lncRNAs and their role in inflammation

LncRNAs	Changes by CS	Functions on inflammation	Mechanism	Disease	References	Year
HOTAIR	Upregulation [100, 101]	Pro-inflammation	Promote TNF-α production by activating NF-κB signaling Promote the expression of protein kinase resource, TNF-α and IL-6 Promote the expression of IL‐6, IL‐1β, COX2, and TNF-α Upregulated by pro-inflammatory cytokine IL-6	Sepsis Keratinocyte injury Atherosclerosis Lung cancer	Wu et al. [201] Liu and Zhang [203] Liu et al. [204] Liu et al. [101]	2016 2018 2019 2015
MALAT1	Upregulation [103]	Pro-inflammation Anti-inflammation	Activate p38 MAPK/NF-κB Increase serum amyloid antigen 3 Upregulated by pro-inflammatory cytokine IL-6 Inhibit the DNA binding activity of NF-κB	Sepsis Diabetes Sepsis N/A	Chen et al. [205] Puthanveetil et al. [206] Zhuang et al. [208] Zhao et al. [207]	2018 2015 2017 2016
CCAT1	Upregulation [102]	Pro-inflammation	Trigger TNF-α, IFN-γ expression by targeting miR-185-3p, meanwhile upregulated by TNF-α	Inflammatory bowel disease	Ma et al. [209]	2019

*CCAT1* colon cancer-associated transcript-1, *HOTAIR* Hox transcript antisense intergenic RNA, *MALAT1* metastasis associated in lung adenocarcinoma transcript 1

## Conclusion

Genetic factors are unable to fully account for the risk and prognosis of CS-related diseases. A growing body of evidence supports the importance of epigenetic alterations in the initiation and development of CS-induced diseases. CS-mediated alterations in DNA methylation, histone modification, and ncRNAs can affect a variety of molecular and cellular processes, such as inflammation, cell cycle arrest, apoptosis, senescence, and autophagy. Based on our review, there is a wealth of evidence in support of the correlation between epigenetic changes and inflammatory responses. In particular, transcription regulation by *NF*-*κB*, a key pro-inflammatory molecule, appears to have a main function in CS-induced epigenetic changes in the mediation of inflammation. However, the role of epigenetic mechanisms in CS-mediated inflammation is very complex and not fully understood. Different changes of epigenetic alterations may play a pro-inflammatory or an anti-inflammatory role through the regulation of target gene expression, thereby participating in the occurrence and development of CS-medicated inflammatory diseases. The recognition of the alterations in DNA methylation, histone modifications, and ncRNAs provides a better understanding of the molecular basis for CS-medicated inflammation. With increasing levels of in-depth research on epigenetic functions, there is no doubt that, in the near future, epigenetic research will lead to the development of epigenomic tools that offer the potential for novel diagnostic and therapeutic agents for CS-medicated disorders.

## Data Availability

All data generated or analyzed during this study are included in this published article.

## Abbreviations

AZA: 5-aza-2′-deoxycytidine
CBP: c-AMP response element binding protein
CCAT1: colon cancer-associated transcript-1
COPD: chronic obstructive pulmonary disease
COX2: cyclooxygenase-2
COX II: cytochrome C oxidase subunit II
CS: cigarette smoke
DNMT: DNA methyl transferase
ERK: extracellular signal-regulated kinase
EZH2: enhancer of zeste homolog 2
HAT: histone acetyltransferase
HDAC: histone deacetylase
HMT: histone methyltransferase
HOTAIR: Hox transcript antisense intergenic RNA
IκB: inhibitory kappa B
IL-1Ra: interleukin-1 receptor antagonist
LCPAT1: lung cancer progression–association transcript 1
lncRNA: long non-coding RNA
MAFbx: muscle atrophy F-box
MALAT1: metastasis associated in lung adenocarcinoma transcript 1
MAPK: mitogen-activated protein kinase
MSK: mitogen- and stress-activated kinase
MUL1: mitochondrial E3 ubiquitin protein ligase 1
MuRF1: muscle ring finger-1 protein
NF-κB: nuclear factor kappa B
NLRP3: Nod-like receptor protein 3
PPAR: peroxisome proliferator-activated receptor
PRMT: protein arginine methyltransferase
SCAL1: smoke and cancer-associated lncRNA1
SIRT: sirtuin
TETs: Tet-eleven translocation proteins
UPS: ubiquitin–proteasome system
USP: ubiquitin-specific protease
